# Versatility of Hybrid OCT and IVUS Imaging Enhances Contrast-Sparing Management of Eruptive Calcified Nodules

**DOI:** 10.1016/j.jscai.2026.105497

**Published:** 2026-06-30

**Authors:** Natali Sorajja, Khady N. Fall, Brian K. Courtney, Megha Prasad

**Affiliations:** a Department of Medicine, NewYork-Presbyterian Hospital/Columbia University Irving Medical Center, New York, New York; b Department of Medicine, Albert Einstein College of Medicine, Bronx, New York; c Sunnybrook Research Institute, University of Toronto, Toronto, Ontario, Canada

**Keywords:** eruptive calcium nodule, hybrid imaging, intravascular imaging, intravascular ultrasound, optical coherence tomography

## Clinical case

**Figure 1:**
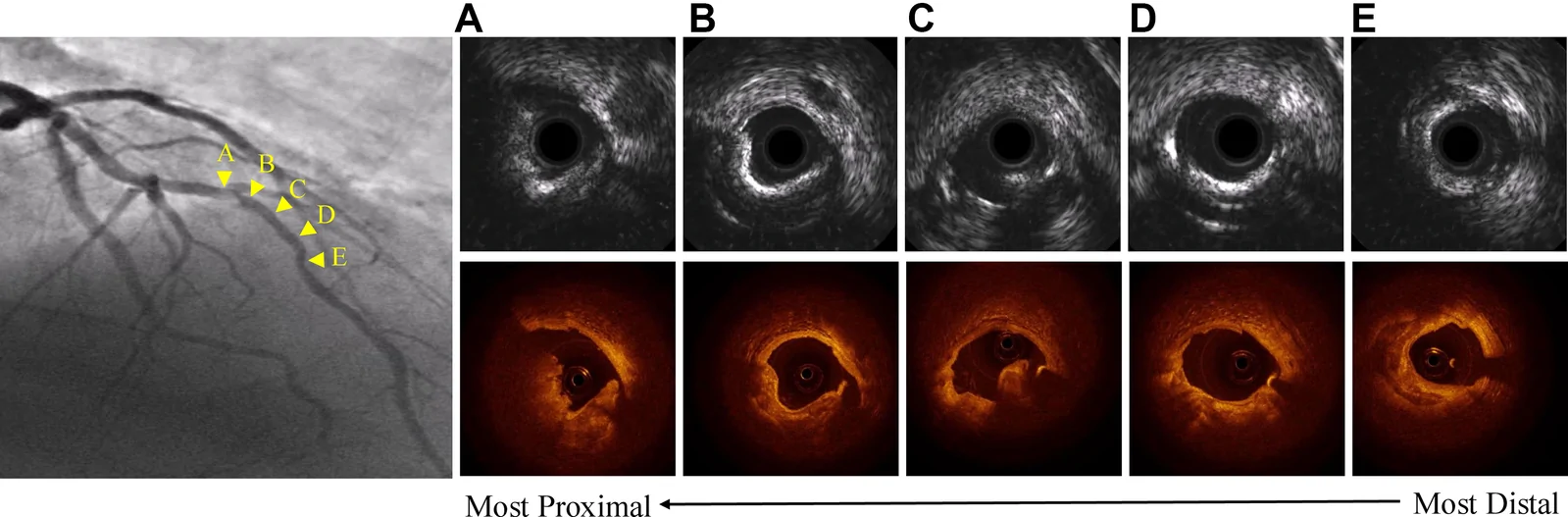
**Angiographic, intravascular ultrasound (IVUS), and optical coherence tomography (OCT) images of the left anterior descending artery.** (**A, B**) Proximal part of the eruptive calcium nodule, where we can measure the minimal lumen area. By IVUS, we can identify at 2 o'clock the second diagonal branch, and we can recognize the calcium nodule protruding with irregular surface also described as jagged edge surface: (**A**) in the lumen from 3 o'clock to 11 o'clock (270°). By OCT, due to higher resolution, we can see a disruption of the fibrous cap at 3 o'clock. The calcium nodule can be misinterpreted as a red thrombus due to the combination of attenuation and protrusion in the lumen. (**B**) There is a dissection flap involving the media from 4 o'clock to 5 o'clock seen by IVUS and even more clear by OCT. Thick calcium plaque (>0.5 mm) contained in the dissection flap. The superficial thick calcium arc extends from 4 o'clock to 10 o'clock. It is followed by a thin calcium arc of <0.5 mm extending from 10 o'clock to 1:00 o'clock. The total calcium arc is ∼270°. This big dissection involving the media needs to be stented because it might lead to an intramural hematoma, which is more frequent and is located between the layers of the media, internal elastic lamina and external elastic lamina. It can also lead to an extramural hematoma, which is between the external elastic lamina and the adventitia. The latter is more severe because these are at higher risk of perforation. (**C**) By IVUS, showing an irregular calcium arc of ∼180°. By OCT, identifying a red thrombus (attenuation) from 3 o'clock to 5:00 o'clock protruding in the lumen. (**D**) By IVUS and OCT, showing a calcium arc from 3 o'clock to 9 o'clock with a lipidic plaque behind the calcium (attenuation by OCT). (**E**) Calcium arc clearly seen by OCT as structure with a well-defined, sharp borders from 6 o'clock to 12 o'clock. Moreover, 180° calcium arc is seen by IVUS.

We present a 64-year-old man with hyperlipidemia, hypertension, impaired renal function (glomerular filtration rate, 42 mL/min/1.73 m^2^), multivessel disease with previous percutaneous coronary intervention (PCI), presenting for staged left anterior descending PCI. The first-generation Novasight Hybrid imaging catheter (Conavi Medical) was used. On initial imaging, an eruptive calcium nodule (ECN) was diagnosed using intravascular ultrasound (IVUS) and optical coherence tomography (OCT). Figure 1 illustrates thrombus overlaying the calcium nodule on OCT, while IVUS clearly delineated the calcium arc.

**Figure 2:**
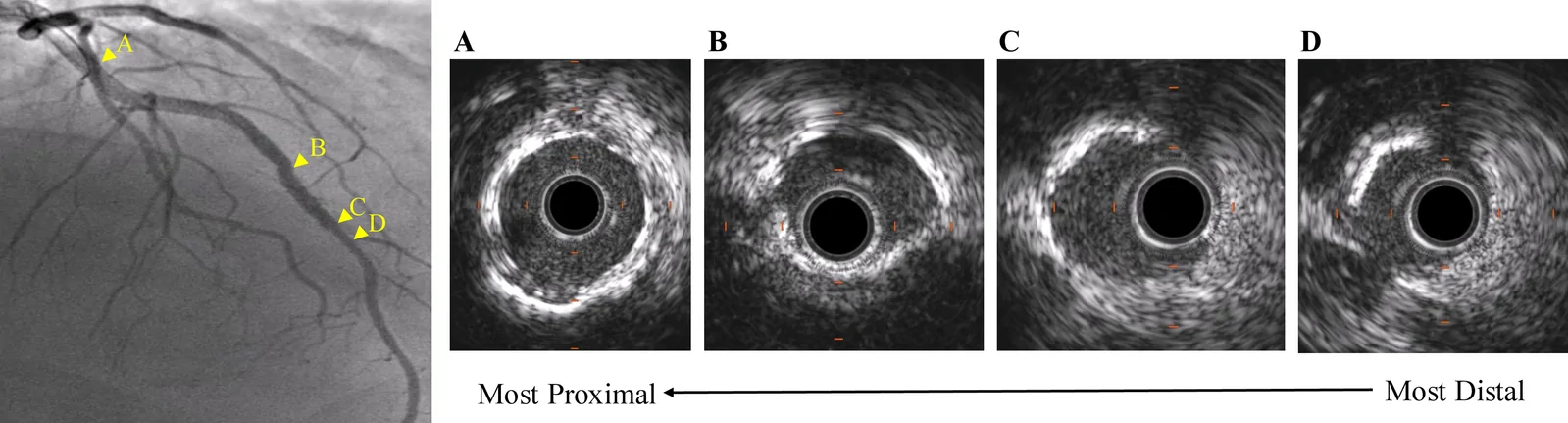
**Post-procedural angiography and intravascular ultrasound imaging.** (**A**) Proximal stent edge. (**B**) Minimal stent area: 7.02 mm^2^. (**C**) Distal stent edge. (**D**) Distal to the stent.

Access to simultaneous coregistered IVUS OCT imaging enhanced ease of diagnosis of ECN while sparing contrast. The jog feature of IVUS alone was used to spare contrast and ensure calcium modification. A final IVUS run was performed to confirm adequate stent expansion and rule out edge dissection (Figure 2). Our case illustrates advantages of simultaneous coregistered dual imaging, allowing flexibility to toggle between hybrid (IVUS-OCT) and IVUS-only pullbacks to diagnose and treat complex lesions while sparing contrast. This system thus allows the operator to leverage advantages of OCT, while not committing to significantly increased contrast usage.

## Procedure

After guide engagement, the Novasight Hybrid imaging catheter was used to perform both an initial angiogram (Supplemental Video 1) and generate hybrid IVUS-OCT imaging to characterize plaque morphology (Supplemental Video 2; Figure 1) using 4 mL of dye. IVUS showed a calcium arc and calcified nodule, while OCT showed some evidence of superficial thrombus (Figure 1). The lesion was predilated with a noncompliant 3.0-mm balloon to 22 atm. No contrast was used thereafter, implementing IVUS-only and jog mode on the catheter. IVUS showed adequate calcium fracture with aggressive balloon dilatation, and 2 stents were delivered and postdilated, confirming adequate stent expansion on IVUS. Thus, OCT was used only for initial guide shot and final image, leveraging IVUS for all remaining parts. A total of 16 mL of contrast was used using this approach, which allowed both contrast limitation with optimal imaging.

## Discussion

This case is of educational value and relevance given the recent designation by the American Heart Association of intravascular imaging (IVI) use (IVUS and OCT) during PCI in acute coronary syndrome (ACS) as a class I recommendation, especially for complex lesions such as ECN.^1^ IVI continues to be underused despite its proven reductions in in-hospital mortality and major adverse cardiovascular events, with commonly cited barriers to adoption often include lack of comfort and expertise in image interpretation.^2^ Hybrid imaging with coregistered simultaneous IVUS OCT may improve the ease with which operators can interpret images and encourage greater utilization. Moreover, it can significantly help reduce contrast exposure while optimizing imaging.

Eruptive calcium nodule is commonly associated with chronic kidney disease (CKD), and challenging to both identify and treat, especially in the presence of thrombus. ECN consists of small, calcified fragments, forming a protruding nodule into the lumen that disrupts the overlying fibrous cap.^3^ ECN may have superficial platelet-rich or red thrombi, which can behave similarly to plaque rupture and thus often present as ACS. ECN account for 2% to 7% of culprit lesions in ACS presentation and is critically important to diagnose on IVI.^4^

Although IVUS and OCT can both discern ECN and calcium arcs individually, each has limitations. OCT can permit measurement of calcium thickness, more sensitive detection of overlying thrombus, and distinction between predominantly red and white thrombus. On OCT, ECN may appear with a lobulated thrombus on top, and, when the thrombus is red, it causes significant signal attenuation and shadowing that can obscure visualization of the underlying nodule. However, OCT may sometimes be challenging to discern a calcium nodule as evidenced by Figure 2A, where the ECN appears as a thrombus. Thus, while OCT can diagnose ECN, it may be misleading when attenuation mimics red thrombus. IVUS, on the contrary, can lack the lateral resolution and contrast to reliably identify superficial thin layers of thrombus. However, IVUS can reliably confirm an arc of calcium via the presence of acoustic shadowing that is usually (but not always) accompanied by a hyperechoic leading edge.^5^ Appreciating the external elastic membrane behind the ECN may be limited using both modalities, as IVUS is subject to overlying thrombus and OCT to light attenuation and lack of penetration depth. The contrast-sparing role of hybrid imaging is useful, allowing use of OCT resolution with IVUS contrast-sparing benefits using a single pullback.

A combined IVUS OCT catheter allows simultaneous coregistered assessment of calcium arc, thickness, vessel size, and presence of red or white thrombus on top of the ECN, while sparing contrast, especially during management of the nodule. Reduction of contrast use is a main advantage of this system and the reason the tool was used. Often, in ACS, renal function is not known until the case has begun, and thus, a contrast-sparing approach can be beneficial. In this case, glomerular filtration rate, was impaired, but we desired OCT for its increased resolution; however, we could not afford excess contrast for an OCT-only approach. The IVUS-only feature was used to reduce contrast. The incidence of CKD is particularly high in patients with calcified nodules.^3^ OCT typically requires flushing of the vessel lumen with contrast media for clear image acquisition, which can contribute toward contrast-induced renal dysfunction, especially in patients with baseline renal impairment. Volume of contrast medium used in OCT-guided procedures has been estimated to be up to 55 mL higher than in IVUS-guided procedures. Thus, many calcified lesions in patients with CKD may not be treated using OCT, limiting use of its higher resolution needed for diagnosis and treatment.

Although IVI has greatly improved our understanding and clinical treatment of coronary artery disease, multimodality imaging catheters may further optimize treatment because of the recognized inherent limitations of IVUS or OCT used individually. This case demonstrates the advantage of hybrid IVUS-OCT imaging to recognize and manage an ECN with limited dye.

## Conclusion

This case of a patient with impaired renal function and an ECN with overlying thrombus illustrates benefits of leveraging hybrid IVUS-OCT imaging, which allowed a contrast-sparing approach of diagnosis and management of an ECN. Hybrid IVUS-OCT imaging may play an important role in patients with challenging plaque morphology, especially in the setting of concomitant CKD.

**Focus Points**

1. A hybrid IVUS-OCT catheter offers flexibility to the operator to use OCT as needed, allowing for a contrast sparing approach while leveraging the benefits of IVUS and OCT individually.
2. Single modality imaging for the diagnosis of ECN may be challenging because of the limitations of IVUS and OCT individually, especially in the setting of impaired renal function.
3. A hybrid IVUS-OCT catheter allows simultaneous coregistered assessment of calcium arc, thickness, vessel size, and presence of red or white thrombus on top of the ECN, which may aid in making the diagnosis.

## CRediT authorship contribution statement

**Natali Sorajja:** Writing – review & editing, Writing – original draft, Visualization, Resources. **Khady N. Fall:** Writing – review & editing, Writing – original draft, Visualization, Supervision, Data curation, Conceptualization. **Brian K. Courtney:** Writing – review & editing, Writing – original draft, Data curation, Conceptualization. **Megha Prasad:** Writing – review & editing, Writing – original draft, Supervision, Conceptualization.

## Declaration of competing interest

Khady N. Fall served as a consultant/speaker for Boston Scientific, Abbott, Conavi, Infraredx, and Terumo. Brian Courtney is a cofounder and minority shareholder of Conavi Medical Inc; receives royalties for patents related to the Novasight Hybrid System; and is a consultant for the Educational Honorarium from ACIST Medical. Brian Courtney’s laboratory receives research funding from Conavi. Brian Courtney has no other affiliation with Conavi. Megha Prasad is a consultant/speaker for Boston Scientific, Abbott Vascular, Boehringer Ingelheim, Chiesi, Conavi, Terumo, and Shockwave Medical. Natali Sorajja reported no financial interests.

## Supplementary material

Supplemental Video 1 Video demonstrating pre-PCI angiography of the left anterior descending artery. PCI, percutaneous coronary intervention.

Supplemental Video 2 Slowed video demonstrating pre-PCI hybrid IVUS OCT pullback. IVUS, intravascular ultrasound; PCI, percutaneous coronary intervention; OCT optimal coherence tomography.

## Footnotes

To access the supplementary material accompanying this article, visit the online version of the *Journal of the Society for Cardiovascular Angiography & Interventions* at 10.1016/j.jscai.2026.105497.
